# Inferior Outcomes after Subsequent Revision or Contralateral ACL Reconstruction: A Cross-Sectional Study

**DOI:** 10.1177/23259671261470914

**Published:** 2026-08-31

**Authors:** Ramana Piussi, Rebecca Hamrin Senorski, Johan Högberg, Riccardo Cristiani, Roland Thomeé, Kristian Samuelsson, Eric Hamrin Senorski

**Affiliations:** † Sahlgrenska Sports Medicine Center, University of Gothenburg, Gothenburg, Sweden; ‡ Unit of Physiotherapy, Department of Health and Rehabilitation, Institute of Neuroscience and Physiology, Sahlgrenska Academy, University of Gothenburg, Gothenburg, Sweden; § Section of Sports Medicine, Department of Molecular Medicine and Surgery, Karolinska Institute, Stockholm, Sweden; ‖ Stockholm Sports Trauma Research Center, Sophiahemmet, Stockholm, Sweden; ¶ Sportrehab Sports Medicine Clinic, Gothenburg, Sweden; # Department of Orthopaedics, Institute of Clinical Sciences, Sahlgrenska Academy, University of Gothenburg, Gothenburg, Sweden; Investigation performed at Sahlgrenska Sports Medicine Center, University of Gothenburg, Gothenburg, Sweden

**Keywords:** knee, injury, patient-reported outcomes, strength

## Abstract

**Background:**

Outcomes after revision anterior cruciate ligament (ACL) reconstruction (ACLR) have been reported as inferior to those after primary reconstruction, but evidence comparing revision and contralateral ACLR in physical activity and patient-reported outcomes (PROs) during early rehabilitation remains limited.

**Purpose:**

To describe and compare rates of return to preinjury physical activity, muscle strength, and PROs between 12 and 24 months of rehabilitation in patients after primary, revision, and contralateral ACLR.

**Study Design:**

Cohort study; Level of evidence, 3.

**Methods:**

Patients from a rehabilitation registry who underwent ACLR were included in this cross-sectional study. They were categorized into groups, defined as primary (n = 1317), revision (n = 111), or contralateral (n = 80) ACLR. Return to physical activity was assessed using the Tegner activity scale. Muscle function was measured via isokinetic strength testing. PROs were evaluated using different questionnaires. Outcomes were compared across groups at 12, 18, and 24 months. In addition, subgroup analysis was performed, dividing patients by sex.

**Results:**

At 24 months, a significantly greater proportion of patients in the primary ACLR group had returned to their preinjury physical activity level compared with both the revision and contralateral ACLR groups (43.9% vs 27.1% and 25.7%, respectively). Patients in the primary ACLR group scored higher on different questionnaires compared to the other groups at all follow-up time points. Sex-specific analysis showed significant differences among female patients in future knee self-efficacy and quality of life, favoring primary ACLR.

**Conclusion:**

Revision and contralateral ACLR were associated with lower rates of return to preinjury physical activity levels and less favorable PROs between 12 and 24 months after reconstruction compared with primary ACLR. Differences in muscle strength were limited, and most statistically significant differences had small effect sizes, ranging from η^2^ = 0.007 to 0.056 for continuous outcomes and ω = 0.122 for return to preinjury physical activity, suggesting that the clinical relevance of these findings should be interpreted cautiously.

The rates of a subsequent anterior cruciate ligament (ACL) injury after ACL reconstruction (ACLR) are considered high and range between 8% and 26%.^7,29^ After a subsequent ACL injury, variations in treatment outcomes have been described in the literature. Inferior patient-reported outcomes (PROs), lower rates of return to preinjury levels of sport, lower absolute quadriceps and hamstring muscle strength, and more severe (ie, stage IV) radiological osteoarthritis have been reported for patients treated with revision ACLR compared with patients treated with primary ACLR at 1 to 5 years after surgery.^2,15,16,24^ On the contrary, other studies have reported no differences in knee function measured with the Knee injury and Osteoarthritis Outcome Score (KOOS) or in strength deficits between patients treated with primary or revision ACLR at 1 year after treatment.^13,14^ There are, however, limited data available for comparing outcomes after ACLR with subsequent contralateral ACLR.^13^

Understanding and mapping outcomes after primary ACLR, revision ACLR, and contralateral ACLR may be of use for clinicians, as this knowledge can aid in advising patients regarding their expectations and in tailoring rehabilitation strategies to meet the individual needs of patients. The purpose of this study was to describe and compare rates of return to preinjury physical activity, muscle strength, and PROs between 12 and 24 months of rehabilitation after primary, revision, and contralateral ACLR. The hypothesis was that within 24 months, revision and contralateral ACLR would yield lower rates of return to preinjury activity and inferior PROs than primary ACLR.

## Methods

This longitudinal cross-sectional study used the RECORD (REporting of studies Conducted using Observational Routinely-collected Data) as guidance^6^ and was performed with data from the Swedish rehabilitation outcome registry: Project ACL. Project ACL offers continuous and structured evaluations of muscle strength and PROs. The results collected from evaluations are shared with the patients and responsible health care professionals.^17^ Evaluations are scheduled at 10 weeks; 4, 8, 12, 18, and 24 months; and every fifth year after an ACL injury or ACLR. Participation in Project ACL is voluntary, and informed consent is acquired at the time of registration. Upon registration in Project ACL, patients provide descriptive information such as sex, age, height, and weight to their profile, and body mass index (BMI) is calculated. After ACLR, graft choice is registered by the test leader at the time of the first muscle strength test. Subsequent ACL injuries are registered by the patients or responsible physical therapists. The occurrence of a subsequent ACL injury is then double-checked by Project ACL's staff. Project ACL has obtained ethical approval from the Regional Ethical Review Board in Gothenburg, Sweden (Nos. 265-13 and T023-17) and from the Swedish Ethical Review Authority (Nos. 2020-02501 and 2024-08724-01).

### Patients

**Figure 1.**
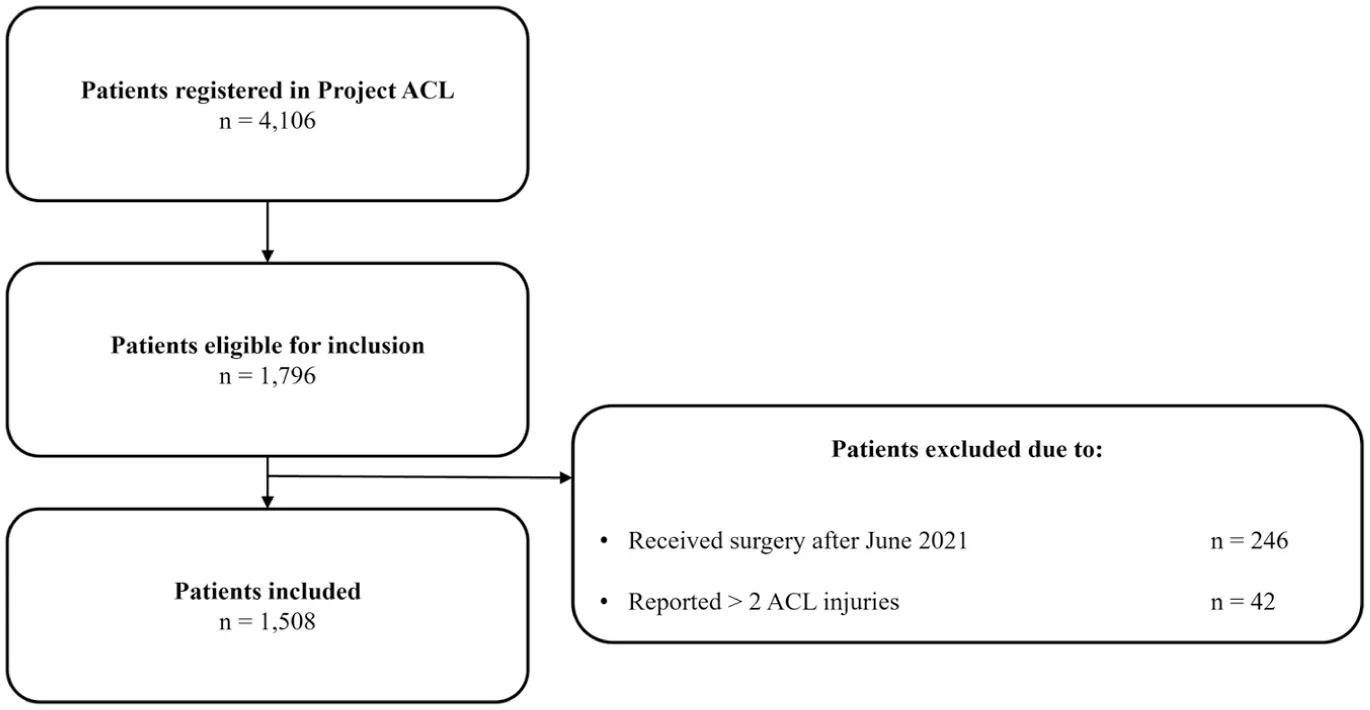
Flowchart of the inclusion and exclusion of patients. ACL, anterior cruciate ligament.

Patients registered in Project ACL, between 2014 and November 2023, who (1) were treated with primary ACLR; (2) were aged between 15 and 50 years at baseline (ie, primary, revision, or contralateral ACLR); (3) reported a preinjury Tegner activity level ≥6; and (4) had available data for at least one of the study outcomes at 12-, 18-, or 24-month follow-up after primary, revision, or contralateral ACLR were eligible for inclusion (Figure 1). Patients who reported more than 2 ACL injuries were excluded. Patients were also excluded if the relevant ACLR procedure had been performed less than 36 months before data extraction in November 2023. The relevant ACLR procedure was defined as primary ACLR for the primary ACLR group, revision ACLR for the revision ACLR group, and contralateral ACLR for the contralateral ACLR group. The 36-month criterion was applied to ensure that sufficient time had elapsed for the scheduled 12-, 18-, and 24-month follow-up visits, with an additional 12-month observation window to identify any further ACL injury before data extraction.

Patients included in this study were divided into 3 groups:

1. The primary ACLR group comprised patients who suffered one ACL injury, treated with ACLR, and did not suffer a second ACL injury for at least 36 months.
2. The revision ACLR group comprised patients treated with primary ACLR who then suffered a second ipsilateral ACL injury, treated with revision ACLR, with no limitation in follow-up time.
3. The contralateral ACLR group comprised patients treated with primary ACLR who then suffered a contralateral ACL injury, treated with ACLR, with no limitation in follow-up time.

Baseline was set to primary ACLR for the primary ACLR group and to revision and contralateral ACLR for the revision and contralateral ACLR groups, respectively. Project ACL includes follow-up visits at 10 weeks and at 4, 8, 12, 18, and 24 months after ACLR. In the present study, analysis was limited to 12-, 18-, and 24-month follow-up because previous review-level evidence suggests that a longer follow-up period may be needed to adequately capture return-to-sport rates.^9^ In addition, return to sport after ACLR has been recommended to be delayed to at least 9 months after surgery, which further supported prioritizing 12-month follow-up and later time points.^21^ The endpoint was set to 24 months after baseline.

Because of the cross-sectional nature of the study, group sizes at follow-up time points were different. In addition, at certain follow-up time points, some patients might have participated in muscle strength testing but did not respond to PRO measures or vice versa. A comparison of characteristics for patients participating at each follow-up time point was performed.

### Outcomes of Interest

The primary outcome of this study was return to preinjury physical activity, defined as returning to the same Tegner activity level as before the injury or higher. Secondary outcomes were results of muscle strength testing and responses to PRO measures: the KOOS Sports and Recreation (Sports) and Quality of Life (QoL) subscales, Knee Self-Efficacy Scale (K-SES) present and future, and ACL–Return to Sport after Injury scale (ACL-RSI), including return to any sport, defined as returning to a Tegner activity level ≥6.

### PRO Measures

At the evaluation time points in Project ACL, patients are invited to complete PRO measures via a web-based platform. The PRO measures included in Project ACL are composed of the KOOS,^31^ the K-SES,^35^ the ACL-RSI,^37^ and the Tegner activity scale.^34^

The KOOS^31^ consists of 42 items rated on 5 subscales: Pain, Symptoms, Activities of Daily Living, Sports, and QoL.^32^ Scores are presented on a 0-to-100 scale, where 0 represents the worst possible subjective knee function and 100 represents the best possible subjective knee function. The 18 items version of the K-SES (K-SES_18_) aims to evaluate knee-related self-efficacy^35^ and comprises 2 subscales: present (14 items) and future (4 items) knee self-efficacy. Scores range from 0 to 10, with worst to best knee-related self-efficacy. The ACL-RSI aims to measure confidence, emotion, and risk appraisal toward return to sport after an ACL injury.^37^ The total score ranges from 10 to 100, worst to best. The Tegner activity scale aims to assess the level of knee-strenuous activity.^34^ The original scale's level ranges from 0 to 10, although Project ACL uses a modified version that starts from level 1.^5^ A higher Tegner activity level indicates higher levels of knee-strenuous activity, where level 6 represents the first level of sole sport participation without work activity as an option.

### Muscle Strength Testing

In Project ACL, testing of muscle strength for knee extension and flexion is performed. All tests are supervised by trained test leaders. Patients are instructed to warm up according to a standardized protocol, which consists of 10 minutes on a stationary bicycle and submaximal trials before each test. Unilateral muscle strength tests are performed using a seated isokinetic dynamometer (System 4; Biodex) between 0° and 90° of knee extension-flexion at an angular velocity of 90 deg/s. Then, 3 to 4 maximum attempts with 40 seconds of rest between each attempt are performed, and the maximal torque (in N·m) is registered in Project ACL's database. The Biodex dynamometer used in Project ACL is calibrated every month.

Results of muscle strength testing were reported both as absolute values, normalized to body weight in kilograms (N·m/kg), and as the limb symmetry index (LSI). The LSI was expressed as a percentage by dividing results from the injured leg with results from the noninjured leg, multiplied by 100. For the contralateral ACLR group, the reference limb was the previously operated limb. Because graft choice was missing for some patients, sensitivity analysis restricted to patients with a known graft choice was performed.

### Statistical Analysis

Statistical analyses were performed with SPSS Statistics for Windows (Version 25.0; IBM). For patient characteristics, means with standard deviations were used for continuous variables, medians with ranges for ordinal variables, and counts with percentages for categorical variables. Comparisons were performed using the chi-square test for categorical variables, 1-way analysis of variance for continuous variables, and the independent-samples *t* test for assessing means between 2 groups of continuous variables. For the primary outcome, the chi-square test was used to compare the rates of patients who returned to preinjury physical activity levels between groups, and the chi-square goodness-of-fit test (Cohen ω) was used to calculate the effect size. The Cohen ω can be interpreted according to the following values: 0.00 < 0.09 = negligible; 0.10 < 0.29 = small; 0.30 < 0.49 = medium; and ≥0.50 = large.^11^ In case of statistically significant differences, a post hoc pairwise *Z* test with the Bonferroni correction was performed. A comparison of secondary outcomes across the 3 groups was performed with 1-way analysis of variance. In case of statistically significant differences, the Tukey post hoc test was performed to verify between which groups the difference was present, and the eta-squared (η^2^) was calculated as a measure of the effect. The η^2^ measures the proportion of the total variance in a dependent variable that is associated with the membership of different groups defined by an independent variable.^30^ The η^2^ ranges between 0 and 1, where 0 explains none of the variance between variables and 1 all of the variance.^30^ Comparisons between groups (primary, revision, and contralateral ACLR) were performed at each follow-up time point. In addition, subgroup analysis was conducted, dividing patients by sex, to assess the heterogeneity of findings across different segments of the study population, as sex has been reported to affect outcomes in patients treated with ACLR.^28^ The alpha level was set to <.05.

## Results

### Patient Characteristics

A total of 1508 patients were included in this study (Figure 1), of whom 1317 were in the primary ACLR group, 111 were in the revision ACLR group, and 80 were in the contralateral ACLR group (Table 1). At baseline, patients in the contralateral ACLR group had a higher BMI compared with patients in the primary and revision ACLR groups (24.5 vs 23.5 and 23.6 kg/m^2^, respectively; *P* = .007; η^2^ = 0.004).

**Table 1:** Patient Characteristics at Baseline*^*a*^*

	Total (n = 1508)	Primary ACLR (n = 1317)	Revision ACLR (n = 111)	Contralateral ACLR (n = 80)	*P* Value
Male sex	687 (45.6)	609 (46.2)	50 (45.0)	28 (35.0)	.145*^*b*^*
Age, y	29.2 ± 8.1	29.2 ± 8.2	28.5 ± 6.7	30.1 ± 7.1	.164*^*c*^*
Height, cm	174.1 ± 9.1	174.2 ± 9.1	173.2 ± 8.8	173.2 ± 9.5	.406*^*c*^*
Weight, kg	71.7 ± 12.3	71.5 ± 12.0	71.3 ± 11.6	73.8 ± 16.3	.262*^*c*^*
BMI, kg/m^2^	23.7 ± 2.9	23.5 ± 2.7	23.6 ± 2.5	24.5 ± 4.7	.007^*c*^
Preinjury Tegner activity level					.282^*b*^
6	122 (8.1)	115 (8.7)	5 (4.5)	2 (2.5)	
7	280 (18.6)	246 (18.7)	17 (15.3)	17 (21.3)	
8	361 (23.9)	309 (23.5)	28 (25.2)	24 (30.0)	
9	497 (33.0)	433 (32.9)	36 (32.4)	28 (35.0)	
10	247 (16.4)	213 (16.2)	25 (22.5)	9 (11.3)	
Missing	1 (0.1)	1 (0.1)	0 (0.0)	0 (0.0)	
Graft choice					.510*^*b*^*
Hamstring tendon	1073 (71.2)	957 (72.7)	67 (60.4)	49 (61.3)	
Bone–patellar tendon–bone	255 (16.9)	222 (16.9)	21 (18.9)	12 (15.0)	
Other tendon	26 (1.7)	20 (1.5)	5 (4.5)	1 (1.3)	
Missing	154 (10.2)	118 (9.0)	18 (16.2)	18 (22.5)	
Time between surgery and second ACL injury,*^*d*^* mo	N/A	N/A	34.8 ± 44.0 [18 (1 to 316)]	39.4 ± 38.2 [30 (–44 to 201)]*^*e*^*	.835*^*f*^*

a Data are shown as n (%) or mean ± SD. ACL, anterior cruciate ligament; ACLR, anterior cruciate ligament reconstruction; BMI, body mass index; N/A, not applicable.

b Chi-square test.

c One-way analysis of variance.

d Data are shown as median (interquartile range) in brackets.

e Some patients in the contralateral group suffered 2 injuries before undergoing ACLR; therefore, the time elapsed between the second injury and surgery is negative. This can happen in case of a partial rupture.

f Independent-samples *t* test.

At 24-month follow-up, the contralateral ACLR group included a lower proportion of male patients than the primary and revision ACLR groups among those who responded to PRO measures (22.9% vs 45.7% and 43.8%, respectively; *P* = .032) (Table 2 and Appendix Table A1). A similar numerical difference, although not significant, was observed among male patients who underwent muscle strength testing (22.7% vs 47.8% and 45.9%, respectively) (Table 2 and Appendix Table A2).

**Table 2:** Patients Participating at Each Follow-up Time Point*^*a*^*

	Primary ACLR	Revision ACLR	Contralateral ACLR
Muscle strength testing			
12 mo	898 (412 [45.9])	73 (32 [43.8])	50 (19 [38.0])
18 mo	540 (244 [45.2])	51 (24 [47.1])	33 (11 [33.3])
24 mo	372 (178 [47.8])	37 (17 [45.9])	22 (5 [22.7])
PRO measures			
12 mo	1081 (491 [45.4])	97 (43 [44.3])	70 (23 [32.9])
18 mo	720 (331 [46.0])	70 (33 [47.1])	39 (12 [30.8])
24 mo	495 (226 [45.7])	48 (21 [43.8])	35 (8 [22.9])

a Data are shown as No. (n [%] of male sex). ACLR, anterior cruciate ligament reconstruction; PRO, patient-reported outcome.

### Return to Preinjury Activity Level

**Figure 2.**
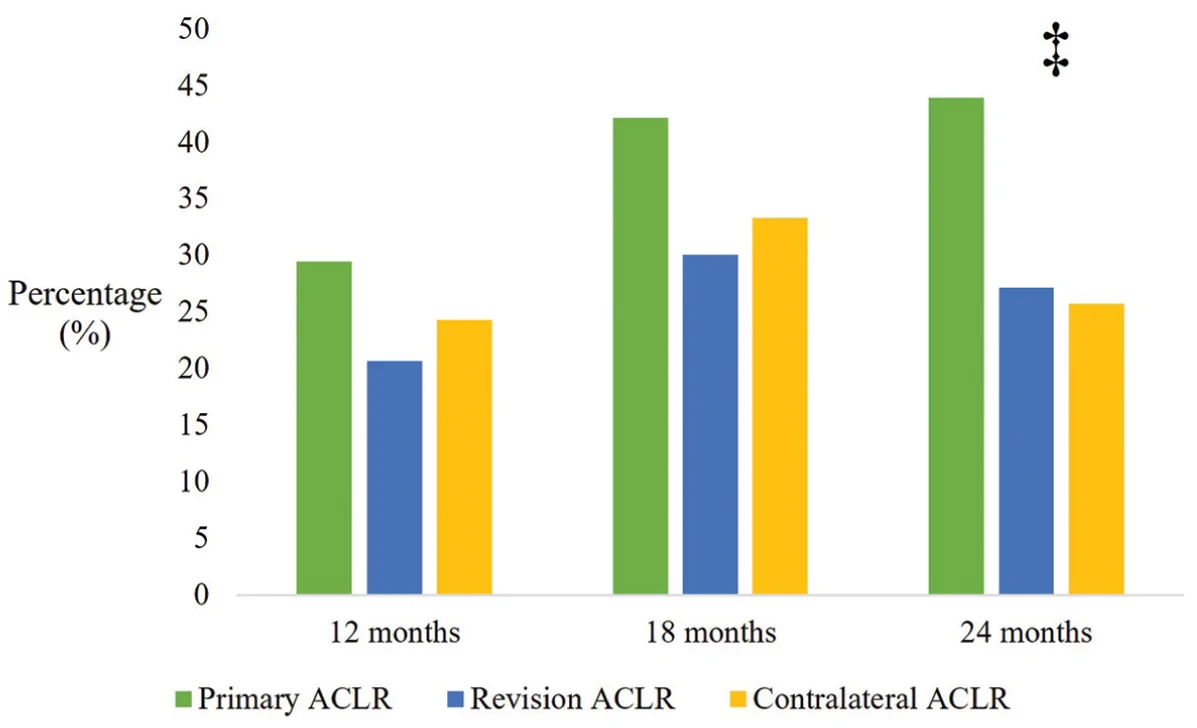
Proportion of patients who reported returning to the same Tegner activity level or higher as before the injury at each follow-up time point. y-axis: percentage of patients. ACLR, anterior cruciate ligament reconstruction. ‡*P* < .05.

At 24 months, a significantly greater proportion of patients in the primary ACLR group had returned to preinjury activity levels compared with both the revision and contralateral ACLR groups (43.9% vs 27.1% and 25.7%, respectively) (Figure 2). No statistically significant differences in return to preinjury physical activity levels were observed between the revision ACLR group and the contralateral ACLR group at any follow-up time point.

### Muscle Strength

**Appendix Figure A1.**
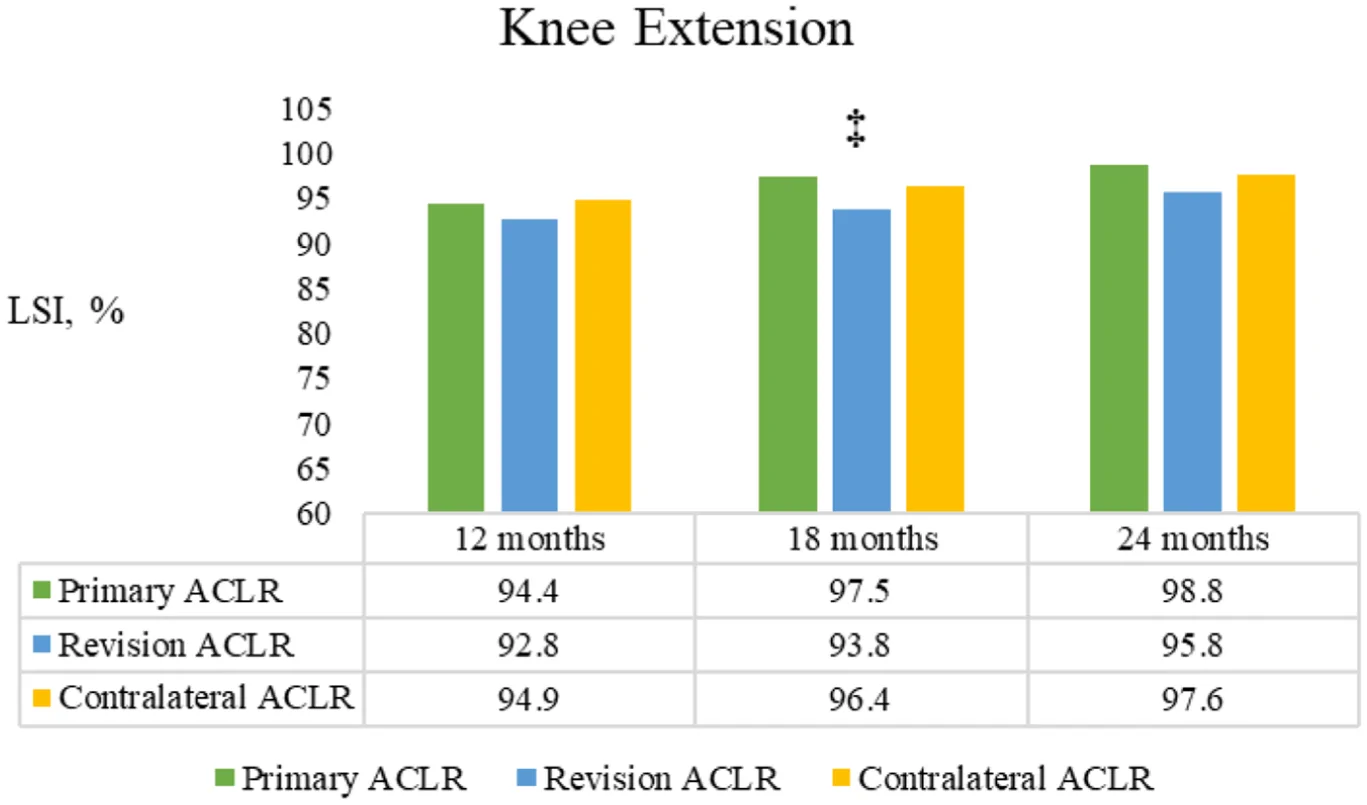
Mean limb symmetry index (LSI) for knee extension for each group and follow-up time point. 18 months: significant difference between primary and revision groups; η^2^ = 0.010. ‡*P* < .05.

**Appendix Figure A2.**
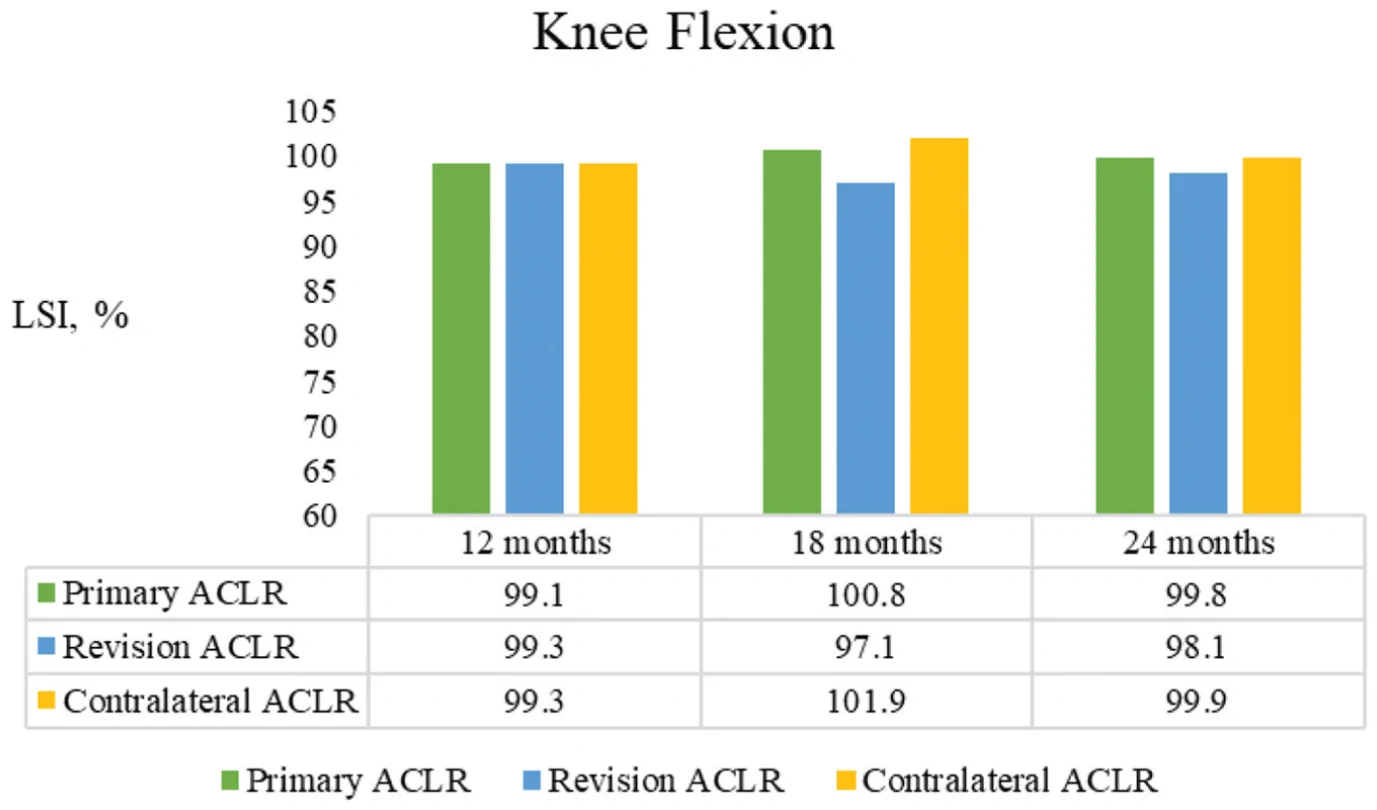
Mean limb symmetry index (LSI) for knee flexion for each group and follow-up time point. ‡*P* < .05.

Patients in the primary ACLR group had a significantly higher knee extension LSI compared to patients in the revision ACLR group at 18 months (97.5 vs 93.8, respectively; *P* = .04; η^2^ = 0.010). However, the difference was below the 10% threshold for clinical relevance^26^ (Appendix Figure A1). No differences were observed for the knee flexion LSI (Appendix Figure A2).

Patients in the revision ACLR group were stronger in knee extension for the noninjured leg at 18 months compared to patients in the primary and contralateral ACLR groups (3.0 vs 2.8 and 2.9 N·m/kg, respectively; *P* = .014; η^2^ = 0.014). No significant differences were found among the groups in knee extension strength for the injured leg or in knee flexion strength for either limb (Appendix Tables A3 and A4).

Because graft choice was missing for some patients, sensitivity analysis restricted to patients with a known graft choice was performed. The pattern of statistically significant findings for all strength outcomes, including the LSI and absolute strength normalized to body weight, was unchanged compared with the results from the main analysis.

### Patient-Reported Outcomes

**Figure 3.**
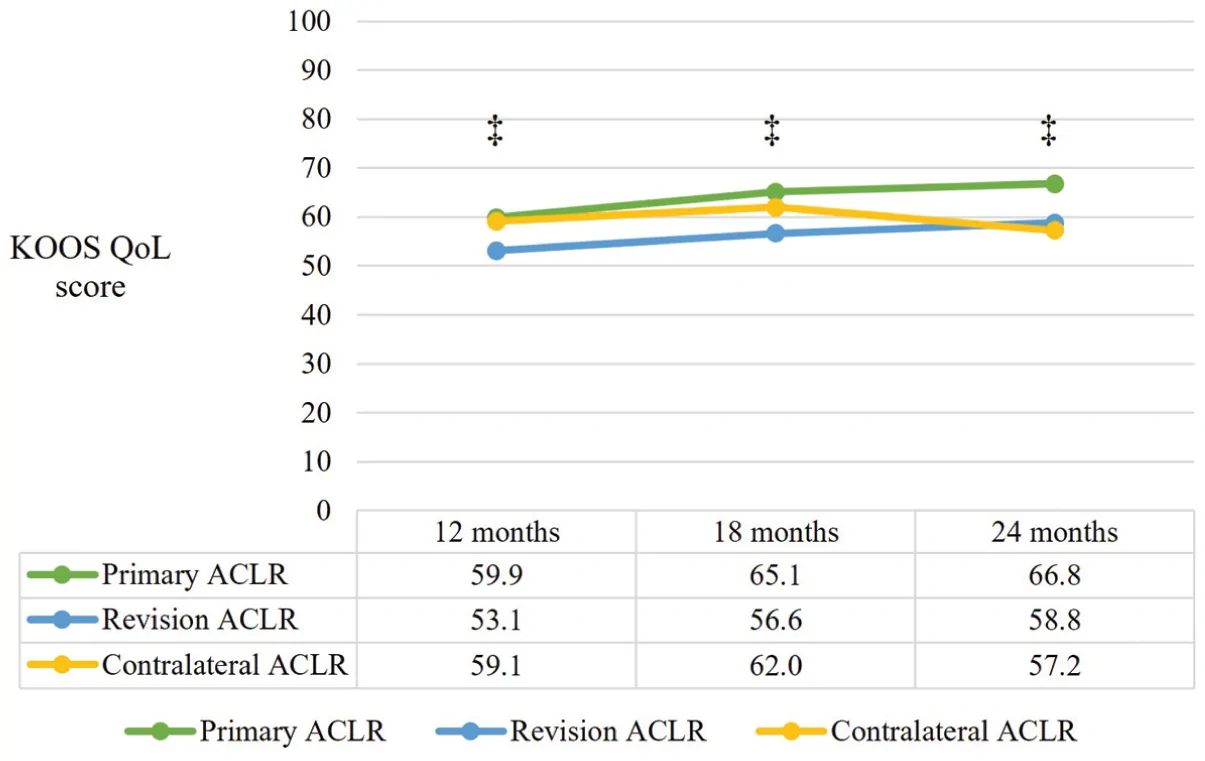
Knee injury and Osteoarthritis Outcome Score (KOOS) Quality of Life (QoL) subscale scores for groups at all follow-up time points. y-axis: KOOS QoL score. ‡*P* < .05.

No significant differences were observed between groups in K-SES present scores or KOOS Sports at any follow-up (Appendix Figures A4 and A5). Patients in the primary ACLR group scored significantly higher on the K-SES future compared with patients in the contralateral ACLR group at 18 months (7.2 vs 6.4, respectively; *P* = .049; η^2^ = 0.012) and at 24 months (7.0 vs 5.8, respectively; *P* = .010; η^2^ = 0.020) (Appendix Figure A6). The primary ACLR group also reported significantly higher KOOS QoL scores compared to the revision ACLR group at 12 months (65.1 vs 56.6, respectively; *P* = .003; η^2^ = 0.009), 18 months (65.1 vs 56.6, respectively; *P* = .002; η^2^ = 0.015), and 24 months (66.8 vs 58.8, respectively; *P* = .029; η^2^ = 0.022) and compared to the contralateral ACLR group at 24 months (66.8 vs 57.2, respectively; *P* = .022; η^2^ = 0.022) (Figure 3).

**Figure 4.**
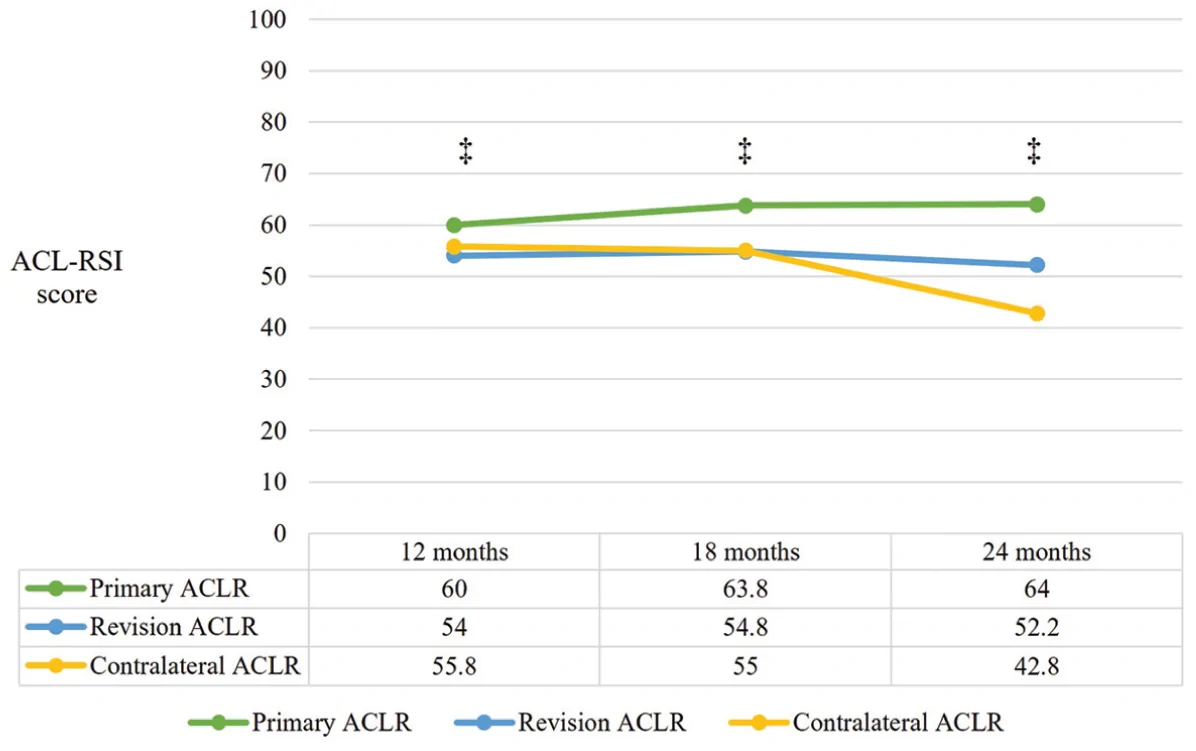
Anterior Cruciate Ligament–Return to Sport after Injury scale (ACL-RSI) scores for groups between 12- and 24-month follow-up. y-axis: ACL-RSI score. ACLR, anterior cruciate ligament reconstruction. ‡*P* < .05.

For the ACL-RSI, patients in the primary ACLR group reported significantly higher scores compared with patients in the revision ACLR group at 12 months (60.0 vs 54.0, respectively; *P* = .042; η^2^ = 0.007), 18 months (63.8 vs 54.8, respectively; *P* = .007; η^2^ = 0.017), and 24 months (64.0 vs 52.2, respectively; *P* = .005; η^2^ = 0.056). Furthermore, patients in the primary ACLR group scored significantly higher compared with patients in the contralateral ACLR group at 24 months (64.0 vs 42.8, respectively; *P* < .001; η^2^ = 0.056) (Figure 4).

At 24-month follow-up, the contralateral ACLR group showed a noticeable drop in ACL-RSI scores compared with 18-month follow-up (42.8 vs 55.0, respectively). To evaluate whether this decline was caused by systematic dropouts of patients with better earlier outcomes, characteristics and ACL-RSI scores at 12 and 18 months for patients in the contralateral ACLR group who did not respond at 24 months were analyzed. No significant differences were found in descriptive variables or in earlier ACL-RSI scores between responders and nonresponders at 24 months.

### Return to Any Physical Activity

**Appendix Figure A3.**
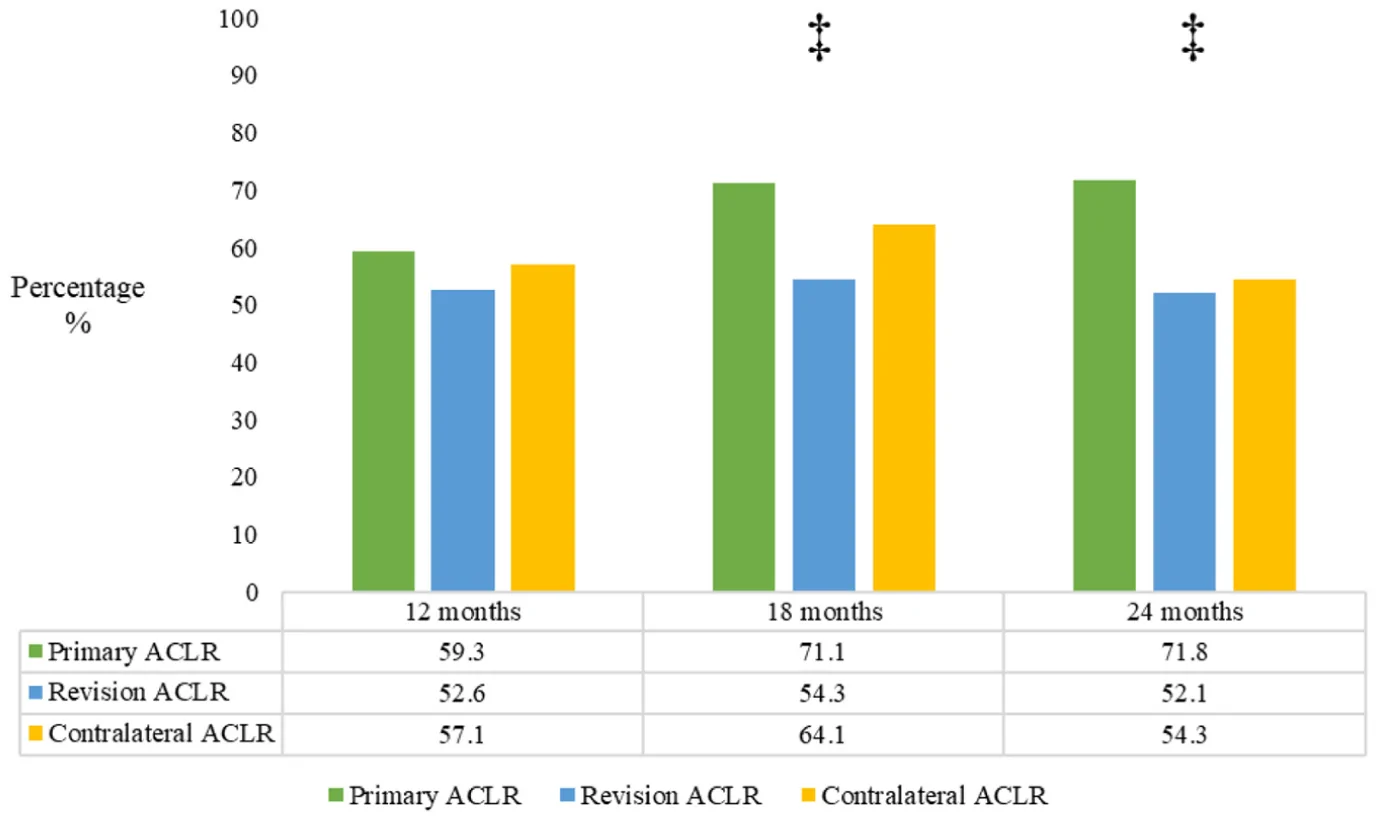
Proportion of patients who reported returning to a Tegner activity level ≥6 at each follow-up time point. ‡*P* < .005.

**Appendix Figure A4.**
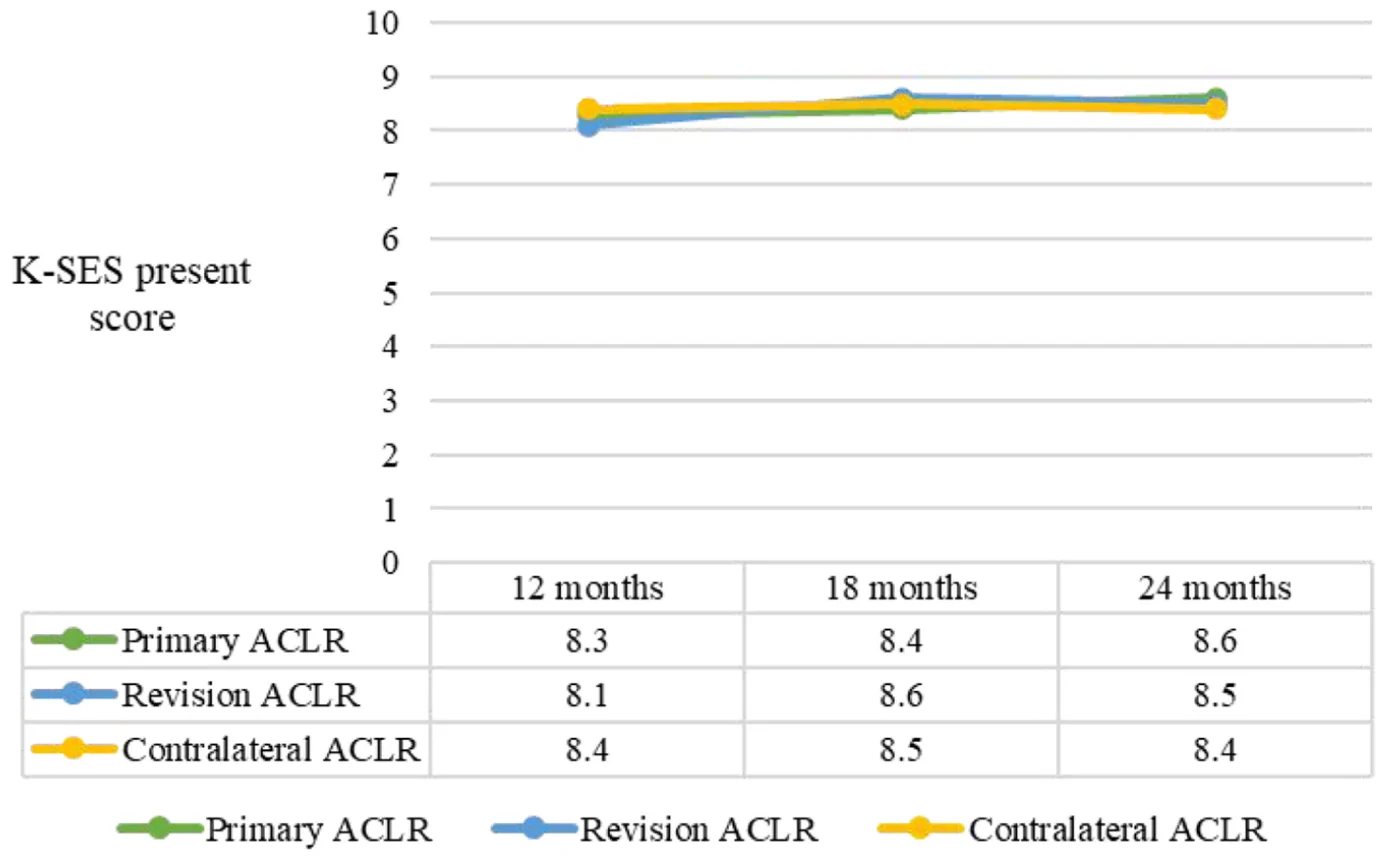
Knee Self-Efficacy Scale (K-SES) present scores for groups at all follow-up time points. ‡*P* < .005.

**Appendix Figure A5.**
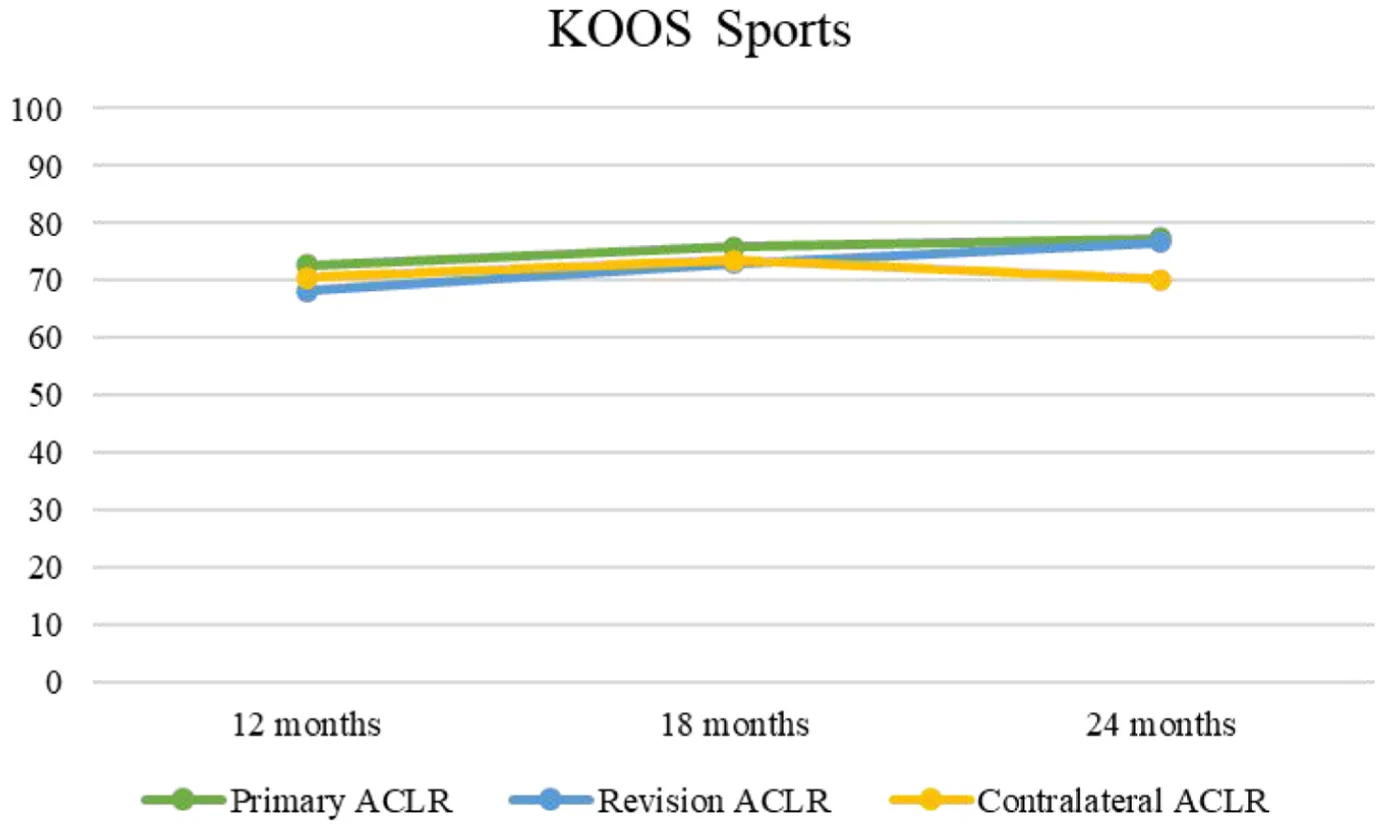
Knee injury and Osteoarthritis Outcome Score (KOOS) Sports subscale scores for groups at all follow-up time points. ‡*P* < .005.

**Appendix Figure A6.**
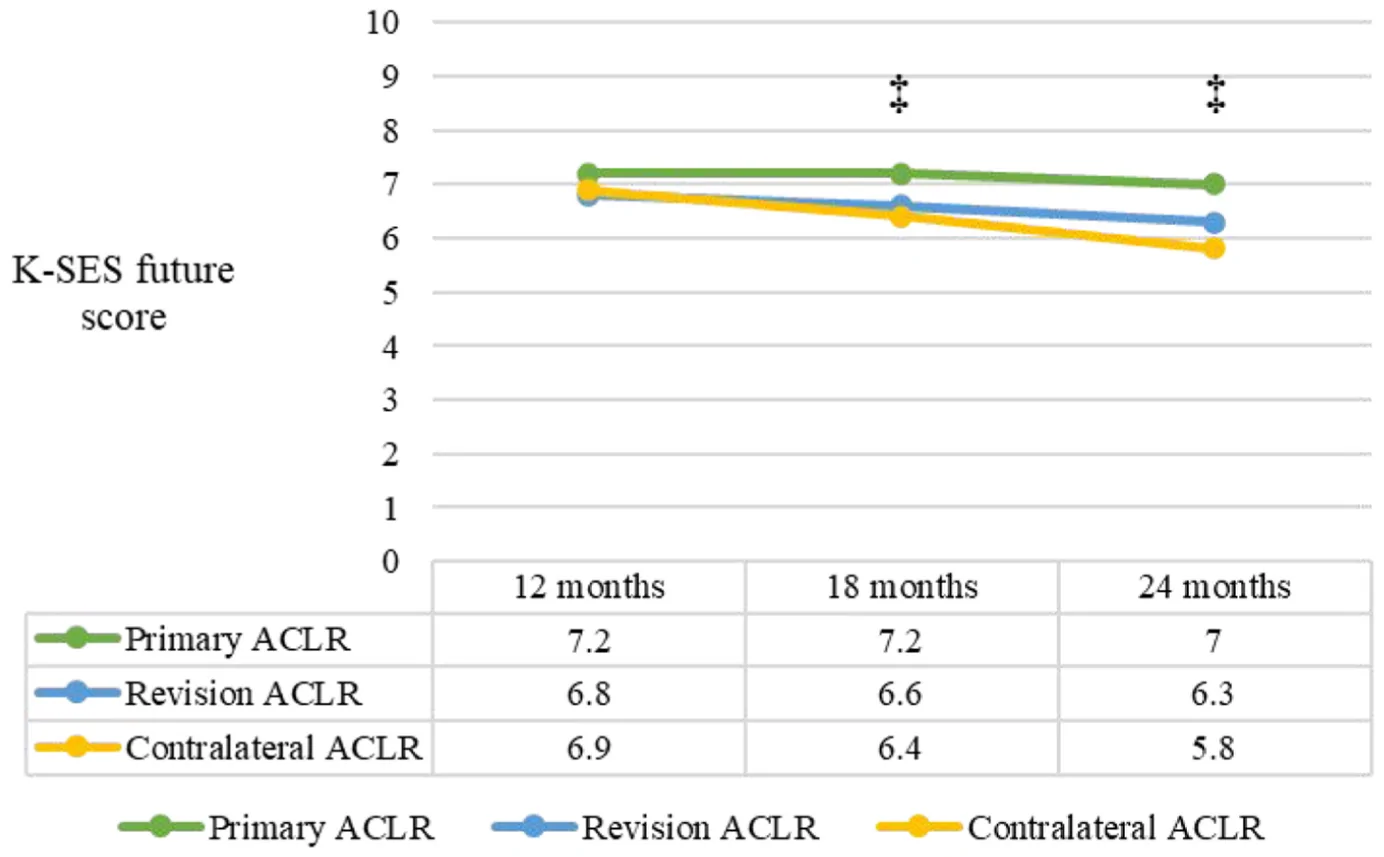
Knee Self-Efficacy Scale (K-SES) future scores for groups at all follow-up time points. ‡*P* < .005.

A greater proportion of patients in the primary ACLR group reported returning to a Tegner activity level ≥6 compared with the revision ACLR group and contralateral ACLR group at 18 months (71.1% vs 54.3% and 64.1%, respectively) and 24 months (71.8% vs 52.1% and 54.3%, respectively) (Appendix Figure A3).

### Subgroup Analysis

Complete subgroup analysis, with outcomes stratified by patient sex, is presented in Supplementary Materials. The female subgroup results mirrored the main analysis for return to preinjury activity levels, future knee self-efficacy, KOOS QoL and ACL-RSI scores. A larger proportion of female patients in the primary ACLR group had a preinjury Tegner activity level of 6 compared with those in the revision and contralateral ACLR groups (11.6% vs 2.9% and 3.1%, respectively; *P* = .007; ω = 0.141).

Among male patients, body weight differed between groups, with a higher body weight in the revision ACLR group compared with both the primary and contralateral ACLR groups (86.1 ± 16.2 vs 80.3 ± 11.2 and 79.7 ± 10.2 kg, respectively; *P* = .004; η^2^ = 0.011). Unlike the main analysis, there were no differences between groups in return to preinjury activity levels, PROs (K-SES future, KOOS QoL, ACL-RSI), or strength at any follow-up time point.

## Discussion

The main finding of this cross-sectional study covering 3 different follow-up time points was that a higher proportion of patients in the primary ACLR group returned to their preinjury or higher physical activity level at 12, 18, and 24 months after surgery compared with both the revision and contralateral ACLR groups, with a statistically significant difference at 24 months (43.9% vs 27.1% and 25.7%, respectively). However, the effect size was small (ω = 0.122). If this holds true in studies involving larger populations, it could suggest that sustaining a new ACL injury in the same or contralateral knee reduces the chance of returning to sport.

### Return to Preinjury Activity Level

The success of rehabilitation after ACLR can be expressed with several outcomes, including return to sport, muscle strength, and PROs. While return to sport is a key goal for many patients, muscle strength and PROs are also considered important to evaluate the rehabilitation progress and to guide treatment strategies.^27,33^ The reported return-to-activity rates after revision ACLR range from 13% to 69%.^16^ In the present study, the highest rate was 43.9%, observed in the primary ACLR group at 24 months. Return to preinjury physical activity is influenced by a range of factors, including physical, psychological, and sociodemographic elements.^10^ This study confirms that suffering 2 ACL injuries impacts the rate of return to preinjury levels of activity. On the other hand, another study reported no differences in activity levels between primary and revision ACLR.^22^ After a second ACL injury, some patients might choose not to return to sport and opt to focus on other aspects in life.^19^ Patient education about potential outcomes, including lower return rates, may further support active engagement in recovery.

### Muscle Strength

At 18 months, the primary ACLR group showed a higher knee extension LSI than the revision ACLR group, suggesting delayed knee extension strength recovery, as previously reported.^8^ There were no differences between the groups in the proportion of patients who received hamstring tendon or bone–patellar tendon–bone autografts.

Although no significant differences were found in absolute knee extension strength (normalized to body weight) for the injured leg, the revision ACLR group was stronger in the noninjured leg at 18 months. Studies have reported that ACLR can lead to varied degrees of knee extension strength recovery, depending on rehabilitation protocols and patient adherence.^4,25^ These findings highlight the importance of individualized rehabilitation that considers injury history, muscle strength status, and personal goals.

### Patient-Reported Outcomes

The primary ACLR group reported significantly higher K-SES future and KOOS QoL scores, as well as emotion, confidence, and risk appraisal measured with the ACL-RSI, compared to the revision and contralateral ACLR groups between 12 and 24 months after baseline. Higher self-efficacy has been associated with better function, pain management, and return to sport after a musculoskeletal injury.^3,36^ One study reported no differences in KOOS subscale scores at 1-year follow-up between patients who suffered a contralateral ACL injury and patients treated with primary ACLR. In contrast, the findings of the present study showed differences in KOOS QoL scores at 12, 18, and 24 months. Patients treated with revision ACLR have been reported to have inferior clinical outcomes and PROs compared with those treated with primary ACLR.^12,20^ This is echoed in qualitative studies describing rehabilitation after a second ACL injury as “lifelong” and marked by persistent symptoms.^19,20^ Collectively, findings from the literature and the present study are important, as they underline the differences in psychological recovery during the rehabilitation process between varying injury types.

### Sex Differences

On sex-stratified analysis, differences between the ACLR groups were observed mainly among female patients, whereas no differences were seen among male patients in return to preinjury activity levels and KOOS QoL, K-SES future, or ACL-RSI scores. Previous data from the Swedish ACL Registry have shown that female patients report inferior PROs compared with male patients after ACLR, supporting the relevance of considering sex in outcome analyses.^1^ Furthermore, female patients are not as physically active as male patients after ACLR.^23^

### Limitations

There are some limitations in this study. First, the data set for this study lacks information on associated injuries, such as meniscal or cartilage lesions. The absence of these data may obscure our understanding of the factors that influence recovery and outcomes after ACL injuries. Second, the validity of the KOOS in this patient population represents another limitation. Research suggests that the KOOS may have lower validity in patients recovering from ACL injuries,^18^ potentially leading to less reliable measurements. Third, the heterogeneity within the contralateral ACLR group, in which both limbs were injured, complicates comparisons across the groups, as the baseline physical condition differs significantly. Another limitation of the present study stems from the extensive number of statistical comparisons conducted, which elevates the risk of a type I error in which false positives could be mistakenly identified as significant findings. To reduce this potential limitation, effect sizes were calculated. All effect sizes for significant results were small or negligible. A major limitation of this study is the low proportion of patients with available follow-up data over time, particularly at 24-month follow-up and in the revision and contralateral ACLR groups. This may have introduced attrition bias, as patients who attended follow-up visits or completed PRO measures may differ systematically from those who did not. For example, patients with better recovery may be less motivated to attend further evaluations, whereas patients with persistent symptoms may either be more likely to seek follow-up or less likely to complete testing. The direction of this potential bias is therefore uncertain. Consequently, the results should be interpreted as reflecting patients with available registry data at each follow-up time point rather than the full eligible population.

Additional registry-related limitations should also be acknowledged. Participation in Project ACL is voluntary, which may have introduced selection bias if patients who registered in the database differed systematically from patients who did not. Furthermore, return to activity was assessed using the Tegner activity scale, which does not account for the frequency or intensity of physical activity, and the registry does not capture whether patients returned to their exact preinjury sport, competitive level, or role within sport. Also, BMI was calculated from self-reported height and weight, which may have introduced measurement errors. Finally, the registry does not capture the exact date of a reinjury or sport type; therefore, age at reinjury, time from reinjury to surgery, and sport-specific analyses could not be explored. Thus, the external validity of the results should be appreciated cautiously.

## Conclusion

Revision and contralateral ACLR were associated with lower rates of return to preinjury physical activity levels and less favorable PROs between 12 and 24 months after reconstruction compared with primary ACLR. Differences in muscle strength were limited, and most statistically significant differences had small effect sizes, ranging from η^2^ = 0.007 to 0.056 for continuous outcomes and ω = 0.122 for return to preinjury physical activity, suggesting that the clinical relevance of these findings should be interpreted cautiously.

## Supplemental Material

sj-docx-1-ojs-10.1177_23259671261470914 – Supplemental material for Inferior Outcomes after subsequent Revision or Contralateral ACL Reconstruction, a cross-sectional study Supplemental material, sj-docx-1-ojs-10.1177_23259671261470914 for Inferior Outcomes after subsequent Revision or Contralateral ACL Reconstruction, a cross-sectional study by Ramana Piussi, Rebecca Hamrin Senorski, Johan Högberg, Riccardo Cristiani, Roland Thomeé, Kristian Samuelsson and Eric Hamrin Senorski in The Orthopaedic Journal of Sports Medicine

## Appendix

Summary of differences for patients who completed PRO measures (Appendix Table A1): At 12 months, patients in the contralateral ACLR group had a significantly higher BMI compared to patients in the revision ACLR group and primary ACLR group (24.6 vs 23.6 and 23.4 kg/m^2^, respectively). At 24 months, the contralateral ACLR group had a statistically lower proportion of male patients compared with the primary and revision ACLR groups (22.9% vs 45.7% and 43.8%, respectively).

**Appendix Table A1:** Differences in Patient Characteristics Between Groups Responding to PRO Measures^*a*^

	12 mo	18 mo	24 mo
Male sex	NS	NS	.032^*b*^
Age, y	NS	NS	NS
Height, cm	NS	NS	NS
Weight, kg	NS	NS	NS
BMI, kg/m^2^	.005^*c*^	NS	NS
Preinjury Tegner activity level			
6	NS	NS	NS
7	NS	NS	NS
8	NS	NS	NS
9	NS	NS	NS
10	NS	NS	NS
Graft choice			
Hamstring tendon	NS	NS	NS
Bone–patellar tendon–bone	NS	NS	NS
Other tendon	NS	NS	NS
Missing	NS	NS	NS

a BMI, body mass index; NS, not significant; PRO, patient-reported outcome.

b Chi-square test.

c One-way analysis of variance.

Summary of differences for patients who participated in muscle strength testing (Appendix Table A2): At 12 months, patients in the primary ACLR group had a significantly lower BMI compared to patients in the contralateral ACLR group (23.4 vs 24.6 kg/m^2^, respectively).

**Appendix Table A2:** Differences in Patient Characteristics Between Groups Participating in Muscle Strength Testing*^*a*^*

	12 mo	18 mo	24 mo
Male sex	NS	NS	NS
Age, y	NS	NS	NS
Height, cm	NS	NS	NS
Weight, kg	NS	NS	NS
BMI, kg/m^2^	.013^*b*^	NS	NS
Preinjury Tegner activity level			
6	NS	NS	NS
7	NS	NS	NS
8	NS	NS	NS
9	NS	NS	NS
10	NS	NS	NS
Graft choice			
Hamstring tendon	NS	NS	NS
Bone–patellar tendon–bone	NS	NS	NS
Other tendon	NS	NS	NS
Missing	NS	NS	NS

a BMI, body mass index; NS, not significant.

b One-way analysis of variance.

Summary of differences for patients in absolute strength normalized to body weight for knee extension (Appendix Table A3): At 18 months, patients in the revision ACLR group had significantly higher knee extension strength in their noninjured limb normalized to body weight compared with patients in the primary ACLR group (3.0 vs 2.8 N·m/kg, respectively).

**Appendix Table A3:** Absolute Strength Normalized to Body Weight for Knee Extension (in N·m/kg)*^*a*^*

	Injured Limb				Noninjured Limb				
	Primary ACLR	Revision ACLR	Contralateral ACLR	*P* Value	Primary ACLR	Revision ACLR	Contralateral ACLR	*P* Value	η^2^ Value
12 mo	2.7	2.7	2.7	NS	2.9	3.0	2.9	NS	
18 mo	2.8	2.9	2.7	NS	2.8	3.0	2.9	.014	0.014
24 mo	2.8	2.8	2.7	NS	2.9	2.9	2.8	NS	

a ACLR, anterior cruciate ligament reconstruction; NS, not significant.

Summary of differences for patients in absolute strength normalized to body weight for knee flexion (Appendix Table A4): There were no differences.

**Appendix Table A4:** Absolute Strength Normalized to Body Weight for Knee Flexion (in N·m/kg)*^*a*^*

	Injured Limb				Noninjured Limb			
	Primary ACLR	Revision ACLR	Contralateral ACLR	*P* Value	Primary ACLR	Revision ACLR	Contralateral ACLR	*P* Value
12 mo	1.5	1.6	1.5	NS	1.5	1.6	1.5	NS
18 mo	1.5	1.5	1.5	NS	1.5	1.4	1.6	NS
24 mo	1.5	1.5	1.5	NS	1.5	1.6	1.5	NS

a ACLR, anterior cruciate ligament reconstruction; NS, not significant.
