# Supplementary material for: Inferior Outcomes after Subsequent Revision or Contralateral ACL Reconstruction: A Cross-Sectional Study
Source: Orthop J Sports Med. 2026 Aug 31;14(8):23259671261470914. doi: 10.1177/23259671261470914 (PMC13530632; doi:10.1177/23259671261470914)
Supplement: Supplementary file 1 [file sj-docx-1-ojs-10.1177_23259671261470914.docx]

Supplementary file 1 for article: …

The differences in statistical significant findings between the entire cohort and the sensitivity analysis performed stratifying patients by sex are marked in red.

When a difference is found, in brackets beside P value the explanation can be found according to the following rule: I = index group; R = revision group, C = contralateral group. The group with the higher/greater score is presented first. For example: I-R = difference where the index group had higher/greater results than the revision group.

Demographics, Women

|  | Entire cohort | Index group | Revision Group | Contralateral Group | P-value | η^2^/ω |
| --- | --- | --- | --- | --- | --- | --- |
| n (%) | 1076 | 942 | 70 | 64 |  |  |
| Age, years | 27.8 (7.7) | 27.9 (7.9) | 26.8 (6.5) | 28.4 (6.3) |  |  |
| Height (cm) | 168.2 (6.1) | 168.2 (6.1) | 168.4 (5.6) | 168.5 (6.5) |  |  |
| Weight (kg) | 65.2 (8.7) | 65.1 (8.8) | 64.6 (8.5) | 66.4 (7.1) |  |  |
| BMI | 22.9 (2.7) | 22.9 (2.7) | 22.7 (2.5) | 23.4 (2.2) |  |  |
| Pre-injury Tegner level |  |  |  |  |  |  |
| 6 | 111 (10.6%) | 107 (11.6%) | 2 (2.9%) | 2 (3.1%) | 0.007 | 0.141 |
| 7 | 178 (16.9%) | 164 (17.8%) | 6 (8.8%) | 8 (12.5%) |  |  |
| 8 | 293 (27.9%) | 246 (26.7%) | 25 (36.8%) | 22 (34.4%) |  |  |
| 9 | 299 (28.4%) | 260 (28.3%) | 17 (25.0%) | 22 (34.4%) |  |  |
| 10 | 171 (16.3%) | 143 (15.5%) | 18 (26.5%) | 10 (15.6%) |  |  |
| Missing | 24 (2.2%) | 22 (2.3%) | 2 (2.8%) | 0 |  |  |
| Graft choice |  |  |  |  |  |  |
| Hamstring n, (%) | 722 (67.1%) | 644 (68.4%) | 42 (60%) | 36 (56.3%) | 0.019 | 0.142 |
| Patella n, (%) | 169 (15.7%) | 144 (15.3%) | 13 (18.6%) | 12 (18.8%) |  |  |
| Other n, (%) | 20 (1.8%) | 16 (1.6%) | 3 (4.3%) | 1 (1.6%) |  |  |
| Missing | 165 (15.3%) | 138 (14.6%) | 12 (17.1%) | 15 (23.4%) |  |  |
|  |  |  |  |  |  |  |
| Time between surgery and second ACL injury (months); mean ± SD; median (min-max) | 33.6 (36.1) |  | 27.9 (32.9)  15 (1-184) | 39.9 (38,6)  30 (-14-201) |  |  |
|  |  |  |  |  |  |  |
| *ACL = Anterior cruciate ligament, BMI = Body mass index, cm = centimetres, kg = kilograms, M = men, n = number, n.a. = not applicable, SD = Standard deviation*  *† chi-square test;*  ** One way ANOVA;*  *⸸ some patients in the contralateral group suffered 2 injuries before receiving an ACL reconstruction: therefore, the time elapsed between second injury and surgery is negative.*  *⸙ independent sample t-test* | | | | | | |

Demographics, Men

|  | Entire cohort | Index group | Revision Group | Contralateral Group | P-value | η^2^ |
| --- | --- | --- | --- | --- | --- | --- |
| n (%) | 1024 | 904 | 76 | 44 |  |  |
| Age, years | 30.5 (7.5) | 30.5 (7.6) | 29.3 (6.4) | 32.2 (6.7) |  |  |
| Height (cm) | 181.2 (6.9) | 181.3 (6.8) | 179.8 (7.2) | 182.5 (7.0) |  |  |
| Weight (kg) | 80.5 (11.4) | 80.3 (11.2) | 86.1 (16.2) | 79.7 (10.2) | 0.004 (R-I; R-C); | 0.011 |
| BMI | 24.4 (3.0) | 24.3 (2.8) | 25.9 (5.6) | 24.6 (2.4) | 0.004 (R-I) |  |
| Pre-injury Tegner level |  |  |  |  |  |  |
| 6 | 60 (6.1%) | 55 (6.4%) | 3 (4.1%) | 2 (4.9%) |  |  |
| 7 | 219 (22.3%) | 195 (22.5%) | 14 (18.9%) | 10 (24.4%) |  |  |
| 8 | 173 (17.7%) | 152 (17.6%) | 14 (18.9%) | 7 (17.1%) |  |  |
| 9 | 352 (35.9%) | 308 (35.6%) | 27 (36.5%) | 17 (41.5%) |  |  |
| 10 | 176 (18%) | 155 (17.9%) | 16 (21.6%) | 5 (12.2%) |  |  |
| Missing | 44 (4.3%) | 39 (4.3%) | 2 (2.6%) | 3 (6%) |  |  |
| Graft choice |  |  |  |  |  |  |
| Hamstring n, (%) | 698 (68.2%) | 624 (69%) | 45 (59.2%) | 29 (65.9%) | 0.011 | 0.150 |
| Patella n, (%) | 165 (16.1%) | 147 (16.3%) | 14 (18.4%) | 4 (9.1%) |  |  |
| Other n, (%) | 20 (2%) | 15 (1.7%) | 4 (5.2%) | 1 (2.3%) |  |  |
| Missing | 141 (13.8%) | 118 (13.1%) | 13 (17.1%) | 10 (22.7%) |  |  |
|  |  |  |  |  |  |  |
| Time between surgery and second ACL injury (months); mean ± SD; median (min-max) | 35.0 (44.9) |  | 32.1 (46.2)  16 (1-316) | 40.1 (42.5)  29 -44-208) |  |  |
|  |  |  |  |  |  |  |
| *ACL = Anterior cruciate ligament, BMI = Body mass index, cm = centimetres, kg = kilograms, M = men, n = number, n.a. = not applicable, SD = Standard deviation*  *† chi-square test;*  ** One way ANOVA;*  *⸸ some patients in the contralateral group suffered 2 injuries before receiving an ACL reconstruction: therefore, the time elapsed between second injury and surgery is negative.*  *⸙ independent sample t-test*  *η2 = eta squared* | | | | | | |

RTS

Women, Return to pre-injury tegner, Yes (%)

|  | Index | Revision | Contralateral | P value | ω |
| --- | --- | --- | --- | --- | --- |
| 4 months | 5 | 5.2 | 2.4 |  |  |
| 8 months | 13.1 | 5.9 | 16.3 |  |  |
| 12 months | 25.8 | 22.2 | 21.3 |  |  |
| 18 months | 41.8 | 32.4 | 25.9 |  |  |
| 24 months | 43.5 | 18.5 | 22.2 | P = 0.006 | 0.175 |

Men, Return to pre-injury tegner, Yes (%)

|  | Index | Revision | Contralateral | P value | ω |
| --- | --- | --- | --- | --- | --- |
| 4 months | 10 | 3.9 | 4.5 |  |  |
| 8 months | 14.2 | 12.5 | 6.5 |  |  |
| 12 months | 33.8 | 18.6 | 30.4 |  |  |
| 18 months | 42.5 | 27.3 | 50 |  |  |
| 24 months | 44.3 | 38.1 | 37.5 |  |  |

Muscle function

LSI Quadriceps

Women

|  | Index | Revision | Contralateral | P value | η^2^ |
| --- | --- | --- | --- | --- | --- |
| 10 weeks | 70.4 | 67.8 | 85.3 | P = 0.000 (C-R) | 0.042 |
| 4 months | 78.7 | 70.2 | 85.2 | P = 0.001 (C-R; C-I) | 0.023 |
| 8 months | 89.1 | 82.7 | 92.4 | P = 0.005 (I-R) | 0.016 |
| 12 months | 94.1 | 91.4 | 94.4 |  |  |
| 18 months | 97.1 | 92.8 | 96.1 |  |  |
| 24 months | 98.5 | 94.1 | 96.7 |  |  |

Men

|  | Index | Revision | Contralateral | P value | η^2^ |
| --- | --- | --- | --- | --- | --- |
| 10 weeks | 73.4 | 74.2 | 85.5 | P = 0.021 (C-I) | 0.016 |
| 4 months | 81.2 | 81.8 | 86.7 |  |  |
| 8 months | 91.1 | 88.3 | 97.4 | P = 0.021 (C-I; C-R) | 0.013 |
| 12 months | 94.8 | 94.6 | 95.6 |  |  |
| 18 months | 97.9 | 95.1 | 97.2 |  |  |
| 24 months | 99.2 | 97.9 | 101.1 |  |  |

LSI Hamstrings

Women

|  | Index | Revision | Contralateral | P value | η^2^ |
| --- | --- | --- | --- | --- | --- |
| 10 weeks | 83.8 | 92.8 | 90.8 | P = 0.001 (R-I) | 0.029 |
| 4 months | 92.9 | 95.1 | 93.9 |  |  |
| 8 months | 96.9 | 96.1 | 96.8 |  |  |
| 12 months | 99.4 | 100.1 | 97.4 |  |  |
| 18 months | 99.7 | 96.6 | 99.5 |  |  |
| 24 months | 100.1 | 99.3 | 99.3 |  |  |

Men

|  | Index | Revision | Contralateral | P value | η^2^ |
| --- | --- | --- | --- | --- | --- |
| 10 weeks | 82.2 | 91.6 | 88.2 | P = 0.004 (R-I) | 0.024 |
| 4 months | 90.9 | 90.8 | 90.7 |  |  |
| 8 months | 96.7 | 95.6 | 101. |  |  |
| 12 months | 98.4 | 98.3 | 102.4 |  |  |
| 18 months | 102.8 | 97.6 | 106.7 |  |  |
| 24 months | 98.8 | 96.4 | 102.1 |  |  |

Muscle function

Absolute values normalized to bodyweight

Women, quadriceps

| Healthy quadriceps | | | | |  |
| --- | --- | --- | --- | --- | --- |
|  | Index | Revision | Contralateral | P value | η^2^ |
| 10 weeks | 2.5 | 2.7 | 2.4 | P = 0.033 (R-C) | 0.013 |
| 4 months | 2.6 | 2.7 | 2.6 |  |  |
| 8 months | 2.6 | 2.8 | 2.7 |  |  |
| 12 months | 2.7 | 2.8 | 2.7 |  |  |
| 18 months | 2.6 | 2.8 | 2.7 |  |  |
| 24 months | 2.6 | 2.7 | 2.6 |  |  |
| Injured quadriceps | | | | |  |
|  | Index | Revision | Contralateral |  |  |
| 10 weeks | 1.7 | 1.8 | 2.0 | P = 0.042 (C-I) | 0.014 |
| 4 months | 2.0 | 1.8 | 2.1 | P = 0.013 (C-R) | 0.014 |
| 8 months | 2.3 | 2.3 | 2.4 |  |  |
| 12 months | 2.5 | 2.5 | 2.5 |  |  |
| 18 months | 2.6 | 2.6 | 2.6 |  |  |
| 24 months | 2.6 | 2.5 | 2.6 |  |  |

Men, quadriceps

| Healthy quadriceps | | | | |  |
| --- | --- | --- | --- | --- | --- |
|  | Index | Revision | Contralateral | P value | η^2^ |
| 10 weeks | 2.9 | 3.0 | 2.8 |  |  |
| 4 months | 3.0 | 3.1 | 2.9 |  |  |
| 8 months | 3.0 | 3.2 | 3.0 |  |  |
| 12 months | 3.0 | 3.1 | 3.0 |  |  |
| 18 months | 3.0 | 3.2 | 3.1 |  |  |
| 24 months | 3.0 | 3.2 | 2.9 |  |  |
| Injured quadriceps | | | | |  |
|  | Index | Revision | Contralateral |  |  |
| 10 weeks | 2.1 | 2.1 | 2.2 |  |  |
| 4 months | 2.4 | 2.4 | 2.5 |  |  |
| 8 months | 2.8 | 2.8 | 2.8 |  |  |
| 12 months | 2.9 | 2.9 | 2.9 |  |  |
| 18 months | 2.9 | 3.0 | 3.0 |  |  |
| 24 months | 3.0 | 3.1 | 2.9 |  |  |

Women, hamstrings

| Healthy hamstrings | | | | |  |
| --- | --- | --- | --- | --- | --- |
|  | Index | Revision | Contralateral | P value | η^2^ |
| 10 weeks | 1.3 | 1.3 | 1.3 |  |  |
| 4 months | 1.2 | 1.3 | 1.3 |  |  |
| 8 months | 1.4 | 1.4 | 1.4 |  |  |
| 12 months | 1.4 | 1.4 | 1.4 |  |  |
| 18 months | 1.4 | 1.4 | 1.3 |  |  |
| 24 months | 1.4 | 1.4 | 1.4 |  |  |
| Injured hamstrings | | | | |  |
|  | Index | Revision | Contralateral |  |  |
| 10 weeks | 1.1 | 1.2 | 1.2 | P = 0.005 (R-I) | 0.022 |
| 4 months | 1.2 | 1.3 | 1.3 |  |  |
| 8 months | 1.3 | 1.3 | 1.3 |  |  |
| 12 months | 1.4 | 1.4 | 1.3 |  |  |
| 18 months | 1.4 | 1.4 | 1.3 |  |  |
| 24 months | 1.4 | 1.3 | 1.4 |  |  |

Men, hamstrings

| Healthy hamstrings | | | | |  |
| --- | --- | --- | --- | --- | --- |
|  | Index | Revision | Contralateral | P value | η^2^ |
| 10 weeks | 1.6 | 1.5 | 1.6 |  |  |
| 4 months | 1.7 | 1.7 | 1.7 |  |  |
| 8 months | 1.7 | 1.8 | 1.7 | P = 0.007 (R-I) | 0.019 |
| 12 months | 1.7 | 1.7 | 1.6 |  |  |
| 18 months | 1.7 | 1.7 | 1.6 |  |  |
| 24 months | 1.7 | 1.8 | 1.6 |  |  |
| Injured hamstrings | | | | |  |
|  | Index | Revision | Contralateral |  |  |
| 10 weeks | 1.3 | 1.5 | 1.3 | P = 0.008 (R-I) | 0.021 |
| 4 months | 1.5 | 1.6 | 1.5 |  |  |
| 8 months | 1.6 | 1.7 | 1.7 |  |  |
| 12 months | 1.6 | 1.7 | 1.6 |  |  |
| 18 months | 1.6 | 1.7 | 1.6 |  |  |
| 24 months | 1.6 | 1.7 | 1.6 |  |  |

PROs

KSES present

Women

|  | Index | Revision | Contralateral | P value | η^2^ |
| --- | --- | --- | --- | --- | --- |
| 10 weeks | 4.0 | 4.1 | 3.8 |  |  |
| 4 months | 5.7 | 5.5 | 5.7 |  |  |
| 8 months | 7.4 | 7.2 | 7.9 |  |  |
| 12 months | 8.2 | 7.8 | 8.4 |  |  |
| 18 months | 8.5 | 8.2 | 8.3 |  |  |
| 24 months | 8.6 | 8.2 | 8.2 |  |  |

Men

|  | Index | Revision | Contralateral | P value | η^2^ |
| --- | --- | --- | --- | --- | --- |
| 10 weeks | 4.5 | 4.1 | 3.9 |  |  |
| 4 months | 6.2 | 6.2 | 6.1 |  |  |
| 8 months | 7.9 | 7.8 | 7.5 |  |  |
| 12 months | 8.4 | 8.4 | 8.4 |  |  |
| 18 months | 8.7 | 8.6 | 8.9 |  |  |
| 24 months | 8.6 | 8.9 | 9.0 |  |  |

KSES future

Women

|  | Index | Revision | Contralateral | P value | η^2^ |
| --- | --- | --- | --- | --- | --- |
| 10 weeks | 7.1 | 6.9 | 6.7 |  |  |
| 4 months | 7. | 6.9 | 6.8 |  |  |
| 8 months | 7.1 | 6.6 | 6.7 |  |  |
| 12 months | 7.2 | 6.4 | 6.8 | P = 0.015 (I-R) | 0.012 |
| 18 months | 7.1 | 6.4 | 6.2 | P = 0.023 (I-R) | 0.017 |
| 24 months | 7.0 | 6.3 | 5.4 | P = 0.001 (I-R) | 0.040 |

Men

|  | Index | Revision | Contralateral | P value | η^2^ |
| --- | --- | --- | --- | --- | --- |
| 10 weeks | 7.3 | 7.0 | 7.3 |  |  |
| 4 months | 7.2 | 7.4 | 6.9 |  |  |
| 8 months | 7.3 | 7.2 | 6.9 |  |  |
| 12 months | 7.2 | 7.2 | 7.2 |  |  |
| 18 months | 7.3 | 6.7 | 6.7 |  |  |
| 24 months | 7.0 | 6.2 | 7.4 |  |  |

KOOS

KOOS Pain

Women

|  | Index | Revision | Contralateral | P value | η^2^ |
| --- | --- | --- | --- | --- | --- |
| 10 weeks | 75.6 | 77.3 | 75.9 |  |  |
| 4 months | 80.2 | 79.5 | 81.2 |  |  |
| 8 months | 85.1 | 82.6 | 86.7 |  |  |
| 12 months | 87.6 | 85.4 | 86.7 |  |  |
| 18 months | 87.9 | 85.2 | 86.3 |  |  |
| 24 months | 89.3 | 88.5 | 88.4 |  |  |

Men

|  | Index | Revision | Contralateral | P value | η^2^ |
| --- | --- | --- | --- | --- | --- |
| 10 weeks | 77.9 | 75.7 | 75.0 |  |  |
| 4 months | 82.1 | 81.6 | 82.1 |  |  |
| 8 months | 86.1 | 86.2 | 83.8 |  |  |
| 12 months | 87.6 | 88.1 | 89.1 |  |  |
| 18 months | 88.8 | 90.3 | 89 |  |  |
| 24 months | 88.4 | 90.6 | 96.8 |  |  |

KOOS Symptoms

Women

|  | Index | Revision | Contralateral | P value | η^2^ |
| --- | --- | --- | --- | --- | --- |
| 10 weeks | 62.4 | 67.3 | 61.3 |  |  |
| 4 months | 70.7 | 70.1 | 72.7 |  |  |
| 8 months | 75.7 | 74.7 | 77.0 |  |  |
| 12 months | 78.9 | 78.5 | 80.8 |  |  |
| 18 months | 79.6 | 77. | 81.0 |  |  |
| 24 months | 82.1 | 75.8 | 82.0 |  |  |

Men

|  | Index | Revision | Contralateral | P value | η^2^ |
| --- | --- | --- | --- | --- | --- |
| 10 weeks | 65.6 | 63.7 | 62.8 |  |  |
| 4 months | 71.5 | 71.9 | 70.1 |  |  |
| 8 months | 75.9 | 77.7 | 75.1 |  |  |
| 12 months | 78.8 | 81.4 | 79.2 |  |  |
| 18 months | 81.1 | 83.8 | 84.5 |  |  |
| 24 months | 81.0 | 85.3 | 86.1 |  |  |

KOOS Sports

Women

|  | Index | Revision | Contralateral | P value | η^2^ |
| --- | --- | --- | --- | --- | --- |
| 10 weeks | 28.7 | 26.6 | 28.5 |  |  |
| 4 months | 47.2 | 43.6 | 46.4 |  |  |
| 8 months | 62.9 | 58.1 | 65.3 |  |  |
| 12 months | 70.9 | 66.3 | 69.1 |  |  |
| 18 months | 73.5 | 69.8 | 71.1 |  |  |
| 24 months | 76.6 | 71.1 | 67.4 |  |  |

Men

|  | Index | Revision | Contralateral | P value | η^2^ |
| --- | --- | --- | --- | --- | --- |
| 10 weeks | 35.4 | 30.9 | 27.6 |  |  |
| 4 months | 51.9 | 52.3 | 44.7 |  |  |
| 8 months | 68.1 | 70.0 | 63.1 |  |  |
| 12 months | 74.8 | 70.5 | 73.5 |  |  |
| 18 months | 78.8 | 76.7 | 79.1 |  |  |
| 24 months | 78.1 | 83.8 | 80.0 |  |  |

KOOS Quality

Women

|  | Index | Revision | Contralateral | P value | η^2^ |
| --- | --- | --- | --- | --- | --- |
| 10 weeks | 37.1 | 37.6 | 37.1 |  |  |
| 4 months | 44.3 | 40.3 | 46.5 |  |  |
| 8 months | 52.8 | 44.6 | 53.2 | P = 0.006 (C-R; I-R) |  |
| 12 months | 59.1 | 51.6 | 58.1 | P = 0.005 (I-R) |  |
| 18 months | 63.7 | 53.4 | 58.7 | P = 0.007 (I-R) |  |
| 24 months | 66.9 | 58.0 | 53.5 | P = 0.001 (I-C) |  |

Men

|  | Index | Revision | Contralateral | P value | η^2^ |
| --- | --- | --- | --- | --- | --- |
| 10 weeks | 38.2 | 37.7 | 35.1 |  |  |
| 4 months | 45.1 | 44.8 | 42.6 |  |  |
| 8 months | 55.1 | 53.6 | 48.1 |  |  |
| 12 months | 61.1 | 55.1 | 61.4 |  |  |
| 18 months | 66.7 | 60.3 | 69.5 |  |  |
| 24 months | 66.7 | 59.9 | 69.7 |  |  |

ACL-RSI

Women

|  | Index | Revision | Contralateral | P value | η^2^ |
| --- | --- | --- | --- | --- | --- |
| 8 months | 52.3 | 53.7 | 55.1 |  |  |
| 12 months | 58.1 | 50.7 | 55.8 |  |  |
| 18 months | 62.5 | 53.7 | 49.8 | P = 0.006 (I-C) | 0.024 |
| 24 months | 63.0 | 50.7 | 37.3 | P = 0.000 (I-C; I-R) | 0.089 |

Men

|  | Index | Revision | Contralateral | P value | η^2^ |
| --- | --- | --- | --- | --- | --- |
| 8 months | 57.1 | 55.9 | 54.7 |  |  |
| 12 months | 62.5 | 58.3 | 56.0 |  |  |
| 18 months | 65.3 | 55.9 | 64.9 |  |  |
| 24 months | 65.2 | 54.2 | 60.5 |  |  |

Return to any physical activity

Women, Return to any physical activity, tegner 6, Yes (%)

|  | Index | Revision | Contralateral | P value | ω |
| --- | --- | --- | --- | --- | --- |
| 4 months | 8.7 | 10.3 | 7.5 |  |  |
| 8 months | 28.2 | 25.5 | 38.8 |  |  |
| 12 months | 55 | 55.6 | 59.6 |  |  |
| 18 months | 68.5 | 51.4 | 66.7 |  |  |
| 24 months | 69.5 | 40.7 | 51.9 | P = 0.003 | 0.187 |

Men, Return to any physical activity, tegner 6, Yes (%)

|  | Index | Revision | Contralateral | P value | ω |
| --- | --- | --- | --- | --- | --- |
| 4 months | 15.2 | 11.8 | 4.5 |  |  |
| 8 months | 37.9 | 35.7 | 45.2 |  |  |
| 12 months | 64.4 | 48.8 | 52.2 |  |  |
| 18 months | 74.2 | 57.6 | 58.3 |  |  |
| 24 months | 74.7 | 66.7 | 62.5 |  |  |
